# Supplementary figures and images for: From movement to METs: A validation of ActTrust® for energy expenditure estimation and physical activity classification in young adults
Source: PLoS One. 2026 May 6;21(5):e0348631. doi: 10.1371/journal.pone.0348631 (PMC13148694; doi:10.1371/journal.pone.0348631)

**A****Male**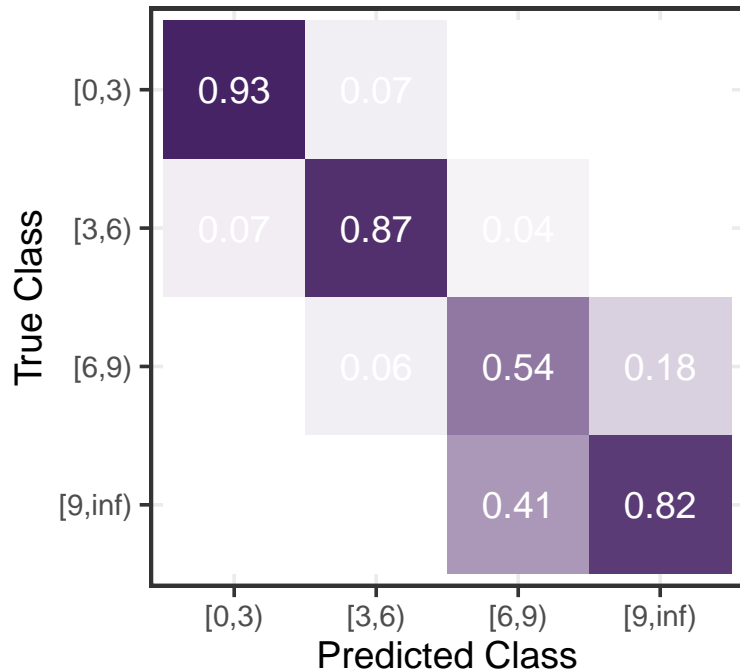**B****Female**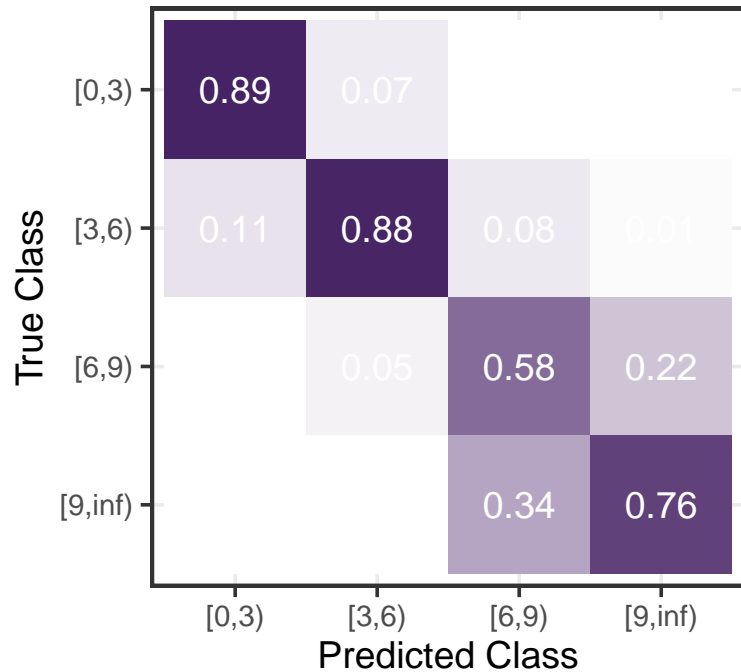

Supplement: S1 Figure — Row-normalised confusion matrices showing the proportion of observations assigned to each predicted intensity class relative to the true class derived from indirect calorimetry for (A) male (n = 34) and (B) female (n = 22) participants. Intensity classes are defined as light [0,3), moderate [3,6), vigorous [6,9), and very vigorous [9,∞) METs. (PDF) [file pone.0348631.s003.pdf]

**A** Normal Q–Q plot

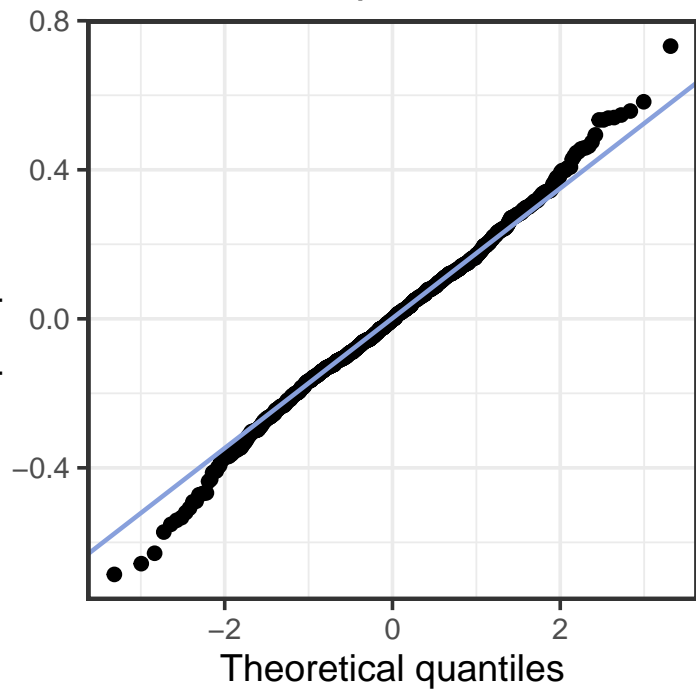

**B** Residuals vs fitted

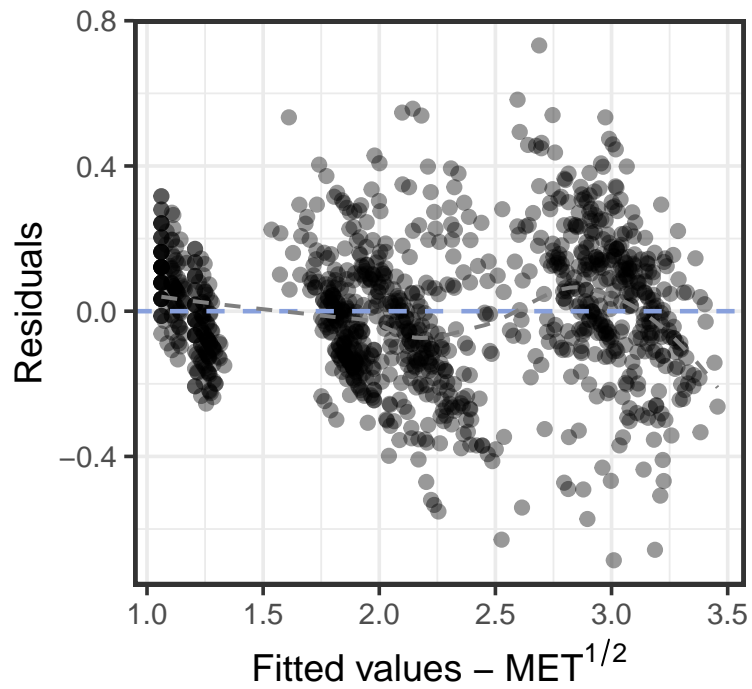

Supplement: S3 Figure — A) Normal Q-Q plot of model residuals with points following the reference blue line, featuring minor deviations at the tails. B) Residuals versus fitted values with no systematic trend supporting the assumption of homoscedasticity. (PDF) [file pone.0348631.s005.pdf]
