# Supplementary material for: From movement to METs: A validation of ActTrust® for energy expenditure estimation and physical activity classification in young adults
Source: PLoS One. 2026 May 6;21(5):e0348631. doi: 10.1371/journal.pone.0348631 (PMC13148694; doi:10.1371/journal.pone.0348631)
Supplement: S2 Figure — Each panel plots the difference between predicted and measured METs against their mean for each device and placement with GT3X + hip (A), GT3X+ wrist (B), ActTrust hip (C), and ActTrust wrist (D). Red dashed lines indicate the mean bias and blue dotted lines indicate the 95% limits of agreement. There is no evidence of systematic proportional bias across the range of measured values. (PDF) [file pone.0348631.s004.pdf]

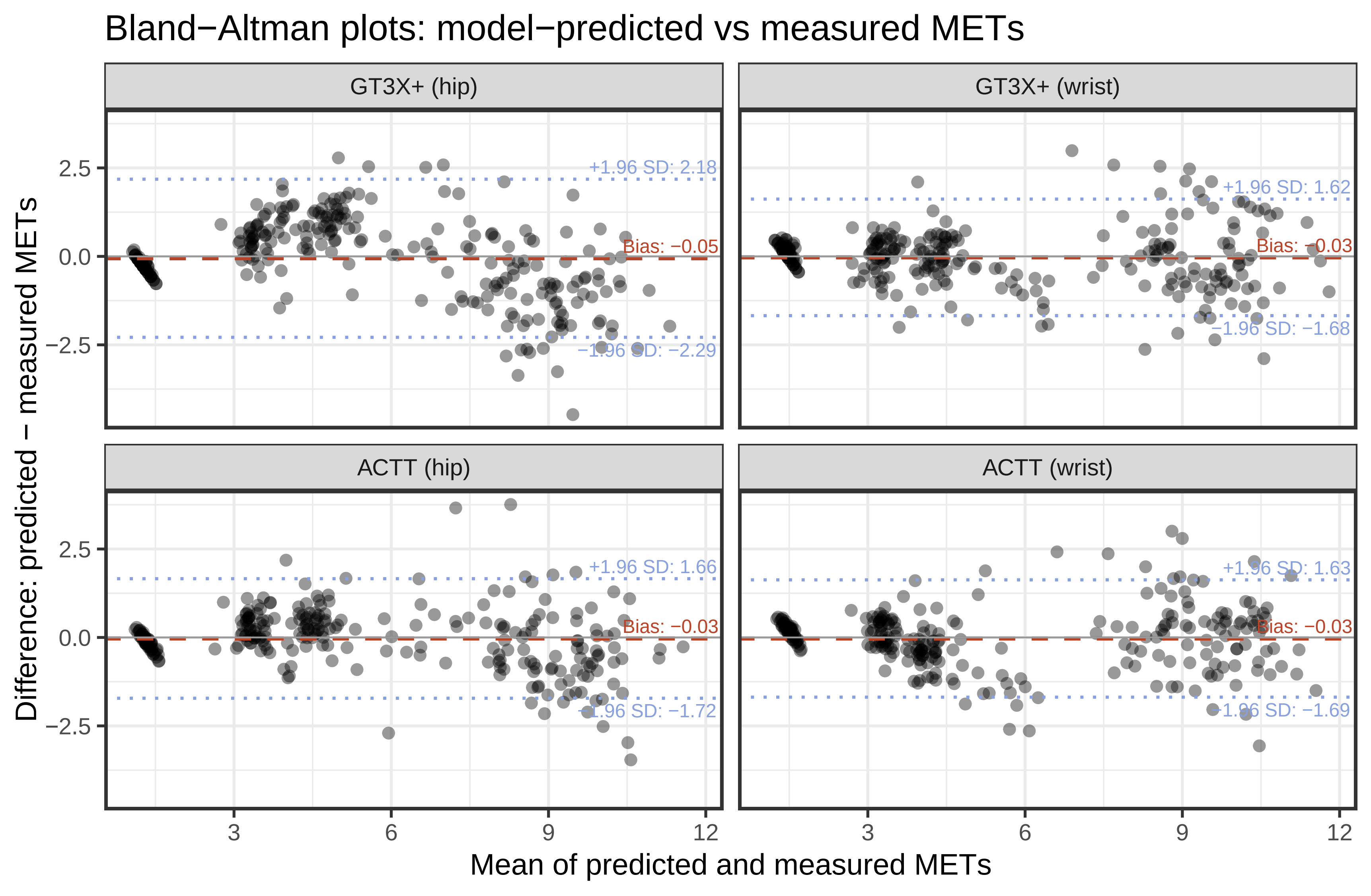

Bland−Altman plots: model−predicted vs measured METs
GT3X+ (hip)
GT3X+ (wrist)
ACTT (hip)
ACTT (wrist)
+1.96 SD: 2.18
Bias: −0.05
−1.96 SD: −2.29
+1.96 SD: 1.62
Bias: −0.03
−1.96 SD: −1.68
+1.96 SD: 1.66
Bias: −0.03
−1.96 SD: −1.72
+1.96 SD: 1.63
Bias: −0.03
−1.96 SD: −1.69
2.5
0.0
−2.5
3
6
9
12
Difference: predicted − measured METs
Mean of predicted and measured METs
