# Supplementary material for: From movement to METs: A validation of ActTrust® for energy expenditure estimation and physical activity classification in young adults
Source: PLoS One. 2026 May 6;21(5):e0348631. doi: 10.1371/journal.pone.0348631 (PMC13148694; doi:10.1371/journal.pone.0348631)
Supplement: Attachment — Submitted filename: Letter .pdf [file pone.0348631.s006.pdf]

Dear researcher,

I received with interest your manuscript submitted to PLOS ONE entitled "**From Movement to METs: A Validation of ActTrust® for Energy Expenditure Estimation and Physical Activity Classification in Young Adults**." It is a relevant, timely, and methodologically sound study in many respects. The validation of accessible and low-cost devices, such as ActTrust®, is crucial for the expansion of physical activity and health research, especially in resource-limited settings. Congratulations on your initiative and the technical rigor employed.

However, as your advisor and reviewer, I feel compelled to point out areas that can and should be improved to increase the article's quality, clarity, scientific rigor, and chances of acceptance. Let's get into the details:

1. Text Structure and Clarity

Positive Points:

The abstract is clear, straightforward, and follows the IMRaD structure (Introduction, Methods, Results, and Discussion) appropriately.

The introduction contextualizes the problem well: the importance of physical activity, the role of METs, the limitations of DLW, and the need for accelerometer validation.

The objective is well defined: to validate ActTrust® and establish cutoff points for activity intensities.

Points for improvement:

Redundancy in the text: There is repetition of sentences between the abstract, introduction, and discussion. For example, the phrase "physical activity is recognized for providing health benefits" appears three times. Avoid this. Each section should have a unique function.

Lack of flow in some paragraphs: Some sections are too dense or poorly connected. Example:

"Accelerometers are not only used to estimate exercise or energy expenditure; they are extremely useful to assess the sleep-wake cycle..."

- This transition is abrupt. Use connectives and transitional paragraphs.
Suggestion: Revise the text with a focus on clarity, conciseness, and logical progression. Use shorter sentences and avoid unnecessary jargon.

2. Methodology

Strong Points:

Sample size (N=56): Adequately justified based on similar studies. Good statistical power.

Control of experimental conditions: Standardized protocol (fasting, resting, fixed speeds, use of an elastic belt).

Use of indirect calorimetry (Quark CPET): Well-applied gold standard.

Preprocessing of respiration data: Exclusion of erratic episodes with a robust criterion (±3 standard deviations).

Data and code availability: Excellent open practice (GitHub). This strengthens reproducibility.

Points for improvement:

Poor justification for choosing 1 second as the epoch:

You state that you used 1 second based on [8], but you don't discuss why this epoch is suitable for all intensities, especially light walking (3 km/h), where movements are smoother.

Problem: Very short epochs increase noise; very long ones lose temporal resolution. Suggestion: Include a brief theoretical justification or cite studies that validated 1s for low-intensity activities.

Lack of detail on synchronization between devices and calorimetry:

How did you synchronize the timing between the metabolism (Quark CPET) and the accelerometers?

Without precise synchronization, there is a risk of temporal misalignment, especially with sudden changes in intensity.

Suggestion: Include a paragraph in "Experimental Procedure" explaining how the synchronization was achieved (e.g., manual timing, triggering, software).

Lack of randomization of the order of conditions:

The protocol always follows: rest → 3 → 5 → 7 → 9 km/h.

This may introduce an order effect (fatigue, learning, warm-up).

Suggestion: State whether the order was fixed and discuss how this may have affected the results (limitation).

3. Statistical Analysis

Strong Points:

Use of two-way ANOVA to compare devices and positions: appropriate.

Generalized linear model with interaction between device and position: well-formulated.

Presentation of robust metrics: sensitivity, specificity, balanced accuracy, AUC.

Points for improvement:

Transformation of the dependent variable (MET^1/2):

You used MET to the square root as the dependent variable.

This is unusual and not justified in the text.

Transformations should be explained: was it to normalize residuals? To increase linearity?

Suggestion: Justify the transformation or test whether the model with linear MET performs as well (or better). If not necessary, remove it.

Regression model with multiple interactions:

The model (Eq. 2) includes complex interaction terms, but there is no test of regression assumptions (normality of residuals, homoscedasticity, multicollinearity).

Suggestion: Include a paragraph in "Data Analysis" about validating the assumptions. Add residual plots in the Supplemental Information.

Low AUC for vigorous activity (0.63):

The AUC for the [3,6] class is 0.63—below the acceptable level (0.7).

This indicates that the model does not classify moderate activity well, despite its high balanced accuracy.

Suggestion: Discuss this in the Discussion as an important limitation, not just a technical finding.

4. Results and Interpretation

Positive Points:

Clear and well-presented figures.

Tables with equations and cutoff points are extremely useful for future researchers.

Confusion matrices are well interpreted.

Points for Improvement:

Claim of "high accuracy" without context:

They say the model has "balanced accuracies above 0.77," which is good, but don't compare it to previous studies.

Suggestion: Compare your cutoff points with those of GT3X+ in the literature (e.g., Santos-Lozano et al., 2013). This provides context.

Claim that the model is "simpler and more explainable":

True, but there is no direct comparison with complex models (e.g., neural networks).

- Suggestion: Rephrase: "Our linear model is simpler and more explainable than black-box models, although we did not directly test alternative models in this study."

5. Discussion

Positive Points:

Good discussion on the importance of integrating sleep and physical activity.

Well-recognized limitations: young sample, controlled environment, few activities.

Areas for Improvement:

Lack of comparison with devices other than the GT3X+:

ActTrust® is new, but could be compared with other low-cost devices (e.g., Fitbit, Garmin).

Suggestion: Briefly mention how ActTrust® positions itself in this ecosystem.

Conclusion Too Optimistic:

They say the device can be used in "clinical practice," but there is no clinical validation.

Suggestion: Soften: "has potential for clinical use, after validation in clinical populations and natural settings."
