# Supplementary material for: From movement to METs: A validation of ActTrust® for energy expenditure estimation and physical activity classification in young adults
Source: PLoS One. 2026 May 6;21(5):e0348631. doi: 10.1371/journal.pone.0348631 (PMC13148694; doi:10.1371/journal.pone.0348631)
Supplement: Attachment — Submitted filename: Response to reviewers.pdf [file pone.0348631.s007.pdf]

Response to Reviewer Comments

Dear Editor,

We thank you and the reviewers for the thorough evaluation of our manuscript "From Movement to METs: A Validation of ActTrust® for Energy Expenditure Estimation and Physical Activity Classification in Young Adults." We are pleased that all reviewers found the study technically sound and clearly written, and we appreciate their constructive feedback for strengthening the manuscript.

We have carefully addressed all comments raised by the four reviewers. The revised manuscript includes: (1) enhanced methodological transparency regarding epoch selection and statistical transformations; (2) addition of sex-stratified analyses and Bland-Altman plots; (3) expanded discussion of ActTrust® advantages and positioning; (4) inclusion of confidence intervals and effect sizes; (5) more thorough comparison with existing literature; and (6) expanded limitations section.

Below, we provide point-by-point responses to each reviewer's comments. All changes are highlighted in the revised manuscript using track changes.

Responses to Editorial Comments:

3. We note that the grant information you provided in the 'Funding Information' and 'Financial Disclosure' sections do not match. When you resubmit, please ensure that you provide the correct grant numbers for the awards you received for your study in the 'Funding Information' section.

Response: The author(s) received no specific funding for this work. We adjusted the manuscript, financial disclosure and funding information accordingly.

4. Please note that your Data Availability Statement is currently missing the repository name and/or the DOI/accession number of each dataset OR a direct link to access each database. If your manuscript is accepted for publication, you will be asked to provide these details on a very short timeline. We therefore suggest that you provide this information now, though we will not hold up the peer review process if you are unable.

Response: Data availability. The data used in the analyses featured in this article are available at https://github.com/circadia-bio/ACTT_validation_study. Code availability. The code used in the analyses featured in this article are available at https://github.com/circadia-bio/ACTT_validation_study. We have checked the information provided to guarantee access to data and code appropriately.

5. Please amend either the abstract on the online submission form (via Edit Submission) or the abstract in the manuscript so that they are identical.

Response: We have matched the information included in the abstract on the online form and in the manuscript.

Responses to Reviewer’s Comments:

Response to Reviewer #1

We thank Reviewer #1 for the positive assessment and valuable suggestions.

Comment 1.1: Gender differences in validation

Reviewer: *Was the gender difference considered for validation? This could be relevant since there is an expected difference between males and females in terms of body composition and energy expenditure, which may act as confounding.*

Response: This is an excellent point. While our primary model pooled males and females together (adjusting for individual differences through the within-subject design), we agree that examining sex-specific validation is important given known differences in body composition and energy expenditure patterns. We have repeated part of our analyses and found similar model performance for both male and female individuals.

Changes Made: We have included confusion matrices and AUC indices separating male and female individuals in supplementary materials.

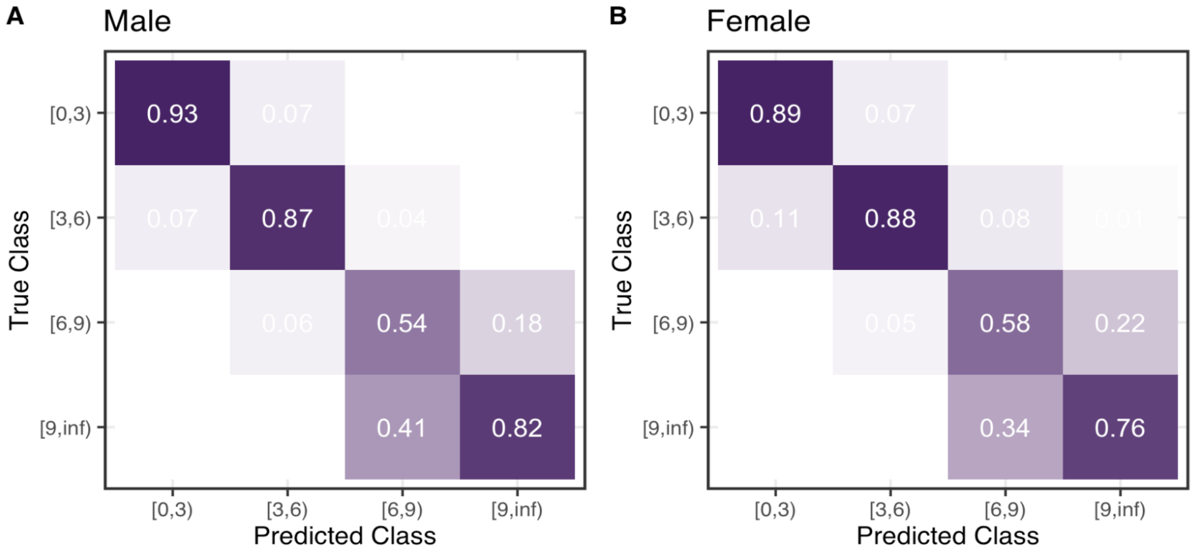

S1 Figure: Confusion matrices for physical activity intensity classification stratified by sex. Row-normalised confusion matrices showing the proportion of observations assigned to each predicted intensity class relative to the true class derived from indirect calorimetry for (A) male and female ($n = 22$) participants. Intensity classes are defined as light [0,3), moderate [3,6), vigorous [6,9), and very vigorous [9,∞) METs.

S2 Table: Area under the curve (AUC) for one-vs-rest physical activity intensity classification stratified by sex. AUC values were computed for each intensity class using the model prediction score against the true class derived from indirect calorimetry for male ($n = 34$) and female ($n = 22$) participants. Intensity classes are defined as light [0,3), moderate [3,6), vigorous [6,9), and very vigorous [9,∞) METs.

Class	Male	Female
[0,3)	0.988	0.974
[3,6)	0.635	0.622
[6,9)	0.765	0.808
[9,∞)	0.961	0.943

Comment 1.2: Bland-Altman plots

Reviewer: *While correlation matrices were reported, additional analysis such as Bland-Altman plots could reveal further details about the systematic bias.*

Response: We thank the reviewer for the suggestion. Additional analyses, included in the supplementary material, demonstrate that there is no evidence of systematic proportional bias across the measured values.

Changes Made: We have created Supplementary Figure S2 showing Bland-Altman plots for each device-placement combination, alongside estimated bias and standard deviation.

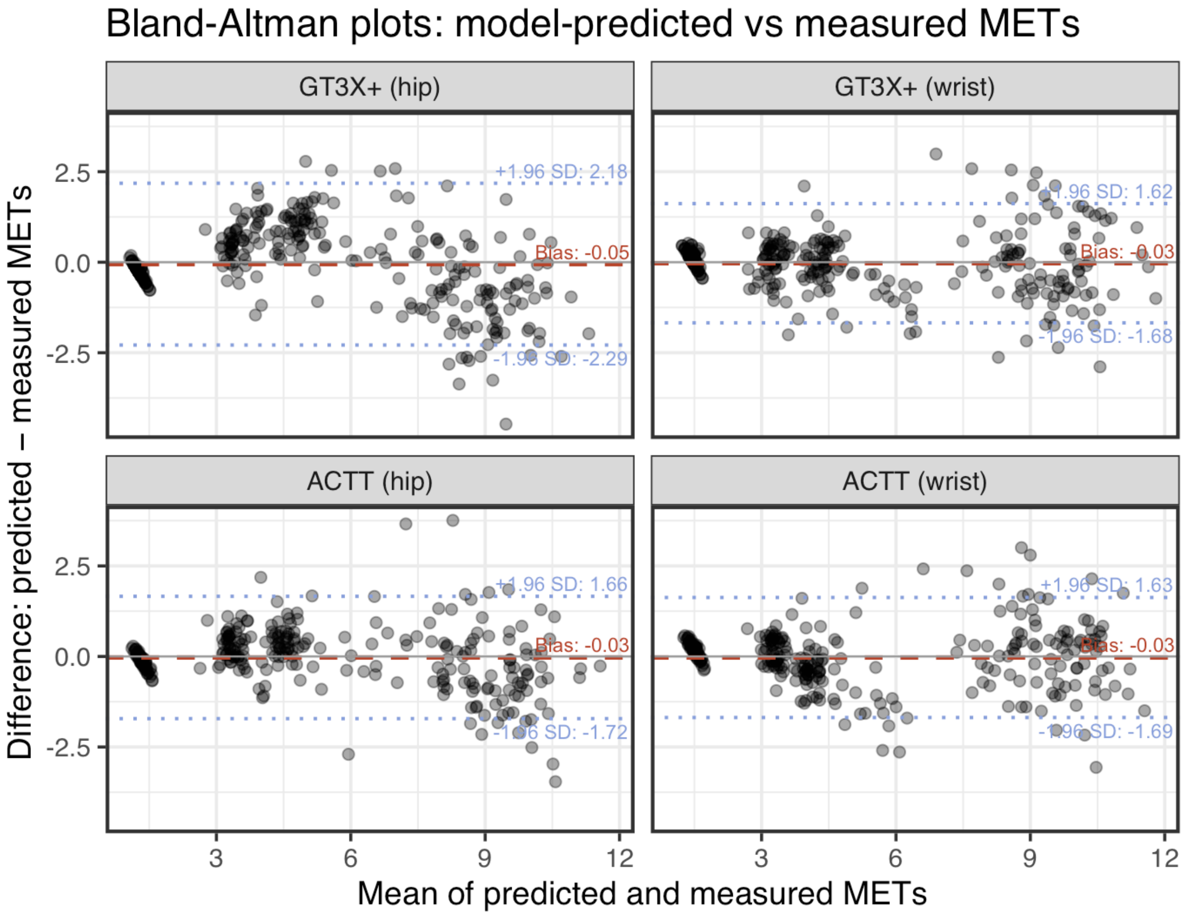

S2 Figure: Bland-Altman plots comparing model-predicted and measured metabolic equivalents (METs) for each device and placement combination. Each panel plots the difference between predicted and measured METs against their mean for each device and placement with GT3X+ hip (A), GT3X+ wrist (B), ActTrust hip (C), and ActTrust wrist (D). Red dashed lines indicate the mean bias and blue dotted lines indicate the 95% limits of agreement. There is no evidence of systematic proportional bias across the range of measured values.

Response to Reviewer #2

We thank Dr. Bueno for the comprehensive and constructive review. We address each point below.

Comment 2.1: Text redundancy

Reviewer: *There is repetition of sentences between the abstract, introduction, and discussion. Each section should have a unique function.*

Response: We appreciate this observation and have reviewed the manuscript accordingly.

Changes made: We removed lines 1-2 abstract and 216-218 (discussion section).

Comment 2.2: Paragraph flow and transitions

Reviewer: *Some sections are too dense or poorly connected with abrupt transitions.*

Response: We have improved paragraph connections and added transitional sentences throughout.

Comment 2.3: Epoch justification

Reviewer: *Poor justification for choosing 1 second as the epoch, especially for light walking (3 km/h).*

Response: We followed epoch size used in literature.

Changes Made: We have clarified that in the manuscript. Lines 87-91.

Comment 2.4: Synchronization details

Reviewer: *Lack of detail on synchronization between devices and calorimetry.*

Response: We performed synchronisation based on timestamps.

Changes made: We detailed the procedure in lines 114-117.

Comment 2.5: Fixed protocol order

Reviewer: *The protocol always follows rest → 3 → 5 → 7 → 9 km/h, which may introduce order effects.*

Response: We acknowledge this limitation. The progressive protocol was chosen for safety and to reflect clinical practice.

Changes Made: We edited experimental procedure (lines 110-111). Moreover, we added to Limitations: " Moreover, the fixed progressive protocol may have introduced order effects such as those linked to warm-up or fatigue. While this approach was chosen for safety, and mirrors clinical exercise testing protocols, future studies could employ counterbalanced designs or include longer rest periods to eliminate carryover effects." (lines 314-317).

Comment 2.6: MET transformation justification

Reviewer: *Transformation of dependent variable (MET^1/2) is unusual and not justified.*

Response: This is a common procedure to linearise data used in many areas of signal processing though not very common in actigraphy. We have clarified that in the manuscript.

Changes Made: We added to the text: "A square root transformation was applied to both METs and activity counts due to the non-linear relationship." (lines 128-129)

Comment 2.7: Regression assumptions

Reviewer: *No test of regression assumptions (normality, homoscedasticity, multicollinearity).*

Response: We conducted these diagnostics but did not include them. Thank you for spotting this issue.

Changes Made: We added to the text: "Model assumptions were verified through examination of residual plots and Q-Q plots for normality – included in supplementary material S3 Figure." (lines 128-131 – data analysis section).

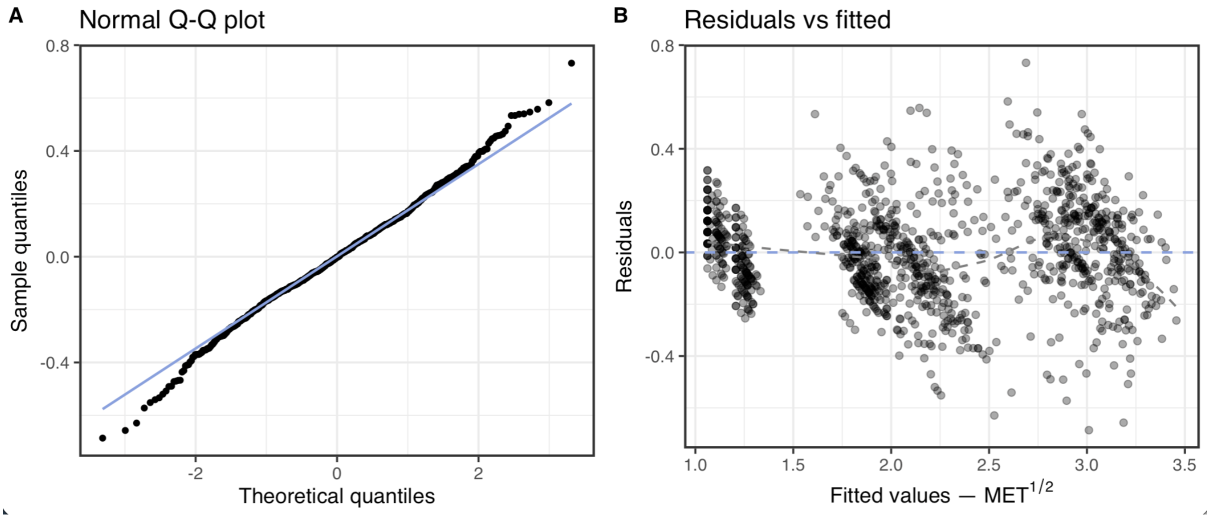

S3 Figure: Regression diagnostics for the general model. A) Normal Q-Q plot of model residuals with points following the reference blue line, featuring minor deviations at the tails. B) Residuals versus fitted values with no systematic trend supporting the assumption of homoscedasticity.

Comment 2.8: Low AUC for moderate activity

Reviewer: *AUC for [3,6] class is 0.63—below acceptable level (0.7). Should be discussed as important limitation.*

Response: Our outcomes are similar to those found in Santos-Lozano A et., 2013. Although we recognise this could be higher, it reflects the heterogeneity and complexity of human movement. We have acknowledged that issue in the discussion.

Changes Made: Added to Discussion: "if we compare our results with studies which used different devices (8,19), while our model's sensitivity and accuracy are reduced for vigorous and very vigorous activity, it still achieves accuracies above 0.90 and higher AUC scores than precious studies. Precisely, our model achieved area under the ROC curve of 0.98, 0.63, 0.78 and 0.95 for light, moderate, vigorous and very vigorous physical activity, respectively. As a comparison, the artificial neural network model developed by Santos-Lozano et al., resulted in AUC of 0.8, 0.6, 0.7 and 0.6 using a very similar protocol (8). (lines 229-236).

Comment 2.9: Context for accuracy claims

Reviewer: *Claim of "high accuracy" without comparison to previous studies.*

Response: We have added explicit comparisons with established literature.

Changes Made: Added to Discussion: "if we compare our results with studies which used different devices (8,19), while our model's sensitivity and accuracy are reduced for vigorous and very vigorous activity, it still achieves accuracies above 0.90 and higher AUC scores than precious studies. Precisely, our model achieved area under the ROC curve of 0.98, 0.63, 0.78 and 0.95 for light, moderate, vigorous and very vigorous physical activity, respectively. As a

comparison, the artificial neural network model developed by Santos-Lozano et al., resulted in AUC of 0.8, 0.6, 0.7 and 0.6 using a very similar protocol (8). (lines 229-236).

Comment 2.10: Model simplicity claim

Reviewer: *Claim about simplicity lacks direct comparison with complex models.*

Response: We clarified our text to facilitate the understanding of our claims. We have added direct comparison with the literature.

Changes Made: "The cut-points derived in the present study for the GT3X+ placed on the hip were lower than most published GT3X reference values across all thresholds. Compared to Sasaki et al. [19] our cut-points were 58%, 21%, and 2% lower at the MET 3, 6, and 9 thresholds, respectively, and 65%, 43%, and 18% lower than Santos-Lozano et al. [8] respective cut-off points for adults. Importantly, however, there is substantial variability in cut-points between these two quite similar studies, as they differ by 16% at MET 3, 39% at MET 6 and 20% at MET 9. The overall variability suggests that sample-level characteristics are the main drivers of the observed discrepancies rather than device or methodological artefacts. Nevertheless, this is the first study to develop movement-based cut-off points for wrist and hip-worn ActTrust® devices. These cut-offs reflect moderate, intense and very intense physical activity categories. The study successfully classified physical activity intensity across the spectrum, demonstrating excellent accuracy. However, it is not possible to compare these values with other studies. For the discrimination between intensity categories based on METs, we propose thresholds to be used for the hip and wrist-worn ActTrust® devices. This allows researchers to examine how much walking people perform and distinguish it from running using these accelerometers." (Lines 241-258).

Comment 2.11: Comparison with other devices

Reviewer: *Lack of comparison with devices other than GT3X+ (e.g., Fitbit, Garmin).*

Response: This is a good suggestion for a future study. We will consider that in the future.

Comment 2.12: Overly optimistic conclusion

Reviewer: *Claim about "clinical practice" without clinical validation.*

Response: We have appropriately softened this claim.

Changes Made: Modified Conclusion: "These results demonstrated the feasibility of using ActTrust® as a cost effective and scalable tool to estimate PA. It also has potential for potential for clinical use following validation in patient populations with diverse health conditions and functional capacities. Our results also provide hints on how to harmonise

outcomes from different devices whilst estimating PA and analysing activity measures. Future work should extend our analyses to free-living conditions, comparison with commercial wearable devices other than GT3X+® and more naturalistic activity patterns." (lines 308-314).

Response to Reviewer #3

We thank Reviewer #3 for highlighting important gaps in our manuscript, particularly regarding the motivation for the study and device benefits.

Comment 3.1: Lack of motivation for experiments

Reviewer: *Why not continue using ActiGraph GT3X+? What benefits does the ActTrust device have?*

Response: This is a crucial point that of our study. We have now clearly articulated the advantages of ActTrust®.

Changes Made: Expanded Introduction: "The ActiGraph® GT3X+ accelerometer has the most significant number of validation studies and published thresholds and has been widely used to validate devices with the same purpose [13]. The ActTrust® (Condor Instruments, São Paulo, Brazil) is a device that has a triaxial accelerometer and is specific for inferring sleep from locomotor activity [14]. It offers several advantages over other actigraphy devices: (1) significantly lower cost, making it accessible for large-scale studies in resource-limited settings; (2) longer battery life enabling extended monitoring periods; (3) integrated light and temperature sensors for comprehensive physiological assessment. However, despite these practical advantages, the ActTrust® has not been validated against gold-standard measures of EE, which limits its use in research and clinical contexts." (lines 25-41).

Comment 3.2: No sleep validation despite device purpose

Reviewer: *The ActTrust device is described as specific for "inferring sleep from locomotor activity," but there is no verification for its use in sleep.*

Response: We understand the confusion. ActTrust® has been validated for sleep assessment in previous studies [43], but never for physical activity intensity classification. Our study fills this gap.

Changes made: "Beyond estimating the intensity of physical activity, the value of validating ActTrust® lies in its ability to capture the entire 24-hour rest-activity cycle within a single device. Therefore, establishing PA cut-points for ActTrust® is a necessary first step toward

leveraging its broader monitoring capabilities, which extend beyond exercise to the assessment of sleep and circadian rhythms. Actigraphy devices, such as the ActTrust accelerometers have been used in sleep and circadian research for decades [41-43]". (lines 259-265).

Comment 3.3: Simultaneous device placement

Reviewer: *Were participants wearing both devices simultaneously? Does location of sensors affect results?*

Response: Yes, participants wore both devices simultaneously (added to line 105). Resulting in four possible combinations of placement and device. As expected the location of sensors does yield different measures in both device types but those seem mostly systematic changes as seen in Fig 2b.

Comment 3.4: Table 1 typo

Reviewer: *Table 1: It says 2*Sample size in the titles. Typo?*

Response: Thank you for catching this formatting error.

Changes Made: Corrected Table 1 header to remove "2*" and properly format the "Sample size" column heading.

Comment 3.5: Gender differentiation in Figure 1

Reviewer: *It would be helpful to differentiate between female and male participants in Figure 1.*

Response: We included an additional analysis in the supplementary materials S1 Figure and S2 Table – see response to Reviewer 1.

Comment 3.6: Cluster in Figure 1B at 7 km/h

Reviewer: *There is a cluster of 10-15 dots at 7 km/h on wrist for both devices. Is there an explanation?*

Response: The explanation of that feature is a matter for a future study. This is probably a issue reported in other studies. These have not added the dots but their error bars increase in that intensity range.

Comment 3.7: Table 2 clarity

Reviewer: *What are the movement counts? 3, 6, 9? Intensity?*

Response: We should have been clearer about what these columns represent.

Changes Made: Revised Table 2 caption to "Model equations and cut-off points for each device and placement combination. Movement counts correspond to thresholds (in counts) for transitions between intensity categories: 3 METs (light to moderate transition), 6 METs (moderate to vigorous), and 9 METs (vigorous to very vigorous)."

Comment 3.8: More thorough results description

Reviewer: *For the results, explain more about each sub-figure instead of just referring to Fig 2.*

Response: We agree that more detailed description would help readers.

Comment 3.9: Figure 2 legend missing "d)"

Reviewer: *Fig 2 legend missing "d)"*

Response: Figure only has 3 panels (a, b, and c)

Comment 3.10: Figure 2C placement

Reviewer: *Fig 2C seems like it would fit better in figure 3.*

Response: Thank you for the suggestion. We believe that Fig 2C belongs in Figure 2.

Comment 3.11: Table 3 lower sensitivity explanation

Reviewer: *Sensitivity in the 2 higher classes are quite a bit lower. AUC is lower in class [3,6). Explanations?*

Response: These are important patterns that deserve discussion. We decided to directly compare our results (AUC) with previous literature. Our outcomes are similar to those found in Santos-Lozano A et., 2013. (lines 229-250).

Comment 3.12: Sleep discussion without sleep data

Reviewer: *A substantial part of the discussion refers to sleep, but there was no explanation why these experiments did not validate this device on sleep.*

Response: We clarify that ActTrust® is already validated for sleep (previous studies), but not for physical activity. Our goal was to complete the validation picture.

Changes made: "Beyond estimating the intensity of physical activity, the value of validating ActTrust® lies in its ability to capture the entire 24-hour rest-activity cycle within a single device. Therefore, establishing PA cut-points for ActTrust® is a necessary first step toward leveraging its broader monitoring capabilities, which extend beyond exercise to the assessment of sleep and circadian rhythms. Actigraphy devices, such as the ActTrust accelerometers have been used in sleep and circadian research for decades [41-43]". (lines 259-265).

Comment 3.13: Expand limitations

Reviewer: *The limitations could be expanded, particularly why results may not be applicable in unhealthy or aged populations.*

Response: Excellent point. We have expanded the limitations section.

Changes Made: Expanded limitations paragraph (lines 299-317).

Response to Reviewer #4

We thank Reviewer #4 for the thorough review and recognition of the study's methodological soundness. We address each major and minor comment below.

Major Comment 4.1: Sample generalizability

Reviewer: *Lack of diversity in age, health status, and functional capacity limits generalizability. Should explicitly acknowledge this and recommend caution.*

Changes Made: We have substantially expanded discussion of generalizability limitations (lines 299-305).

Major Comment 4.2: Statistical model reporting

Reviewer: *Model equations in Table 2 lack confidence intervals or measures of uncertainty (e.g., standard errors).*

Changes Made:

We have revised Table 2 to include 95% confidence intervals for all model parameters.

Major Comment 4.3: Comparison with existing literature

Reviewer: *Need more thorough comparison with existing cut-points from similar devices.*

Response: We have the section about comparison with published thresholds.

Changes Made: "The cut-points derived in the present study for the GT3X+ placed on the hip were lower than most published GT3X reference values across all thresholds. Compared to Sasaki et al. [19], our cut-points were 58%, 21%, and 2% lower at the MET 3, 6, and 9 thresholds, respectively, and 65%, 43%, and 18% lower than Santos-Lozano et al. [8] respective cut-off points for adults. Importantly, however, there is substantial variability in cut-points between these two quite similar studies, as they differ by 16% at MET 3, 39% at MET 6 and 20% at MET 9. The overall variability suggests that sample-level characteristics are the main drivers of the observed discrepancies rather than device or methodological artefacts.

Nevertheless, this is the first study to develop movement-based cut-off points for wrist and hip-worn ActTrust® devices. These cut-offs reflect moderate, intense and very intense physical activity categories. The study successfully classified physical activity intensity across the spectrum, demonstrating excellent accuracy. However, it is not possible to compare these values with other studies. For the discrimination between intensity categories based on METs, we propose thresholds to be used for the hip and wrist-worn ActTrust® devices. This allows researchers to examine how much walking people perform and distinguish it from running using these accelerometers." (lines 241-258).

Major Comment 4.4: Confusion matrix interpretation

Reviewer: *Reduced sensitivity for vigorous and very vigorous activities should be discussed with potential reasons and suggestions for improvement.*

Response: See detailed response to Reviewer #3, Comment 3.11, and Reviewer #2, Comment 2.10, which thoroughly addresses this issue.

Minor Comment 4.5: Abstract numerical results

Reviewer: *Abstract should include key numerical results.*

Response: We have added specific metrics to strengthen the abstract.

Changes Made: Revised abstract to include: "Our results showed correlations between treadmill speed and both METs ($r = 0.95$, $p < 0.05$) and movement counts from both GT3X+ and ActTrust devices placed either on the hip ($r = 0.94$, $p < 0.05$; $r = 0.93$, $p < 0.05$) or on the wrist ($r = 0.88$, $p < 0.05$; $r = 0.88$, $p < 0.05$), respectively."

Minor Comment 4.6: Rationale for 4 central minutes

Reviewer: *Clarify rationale for selecting the 4 central minutes of each condition.*

Response: This was adopted in accordance with previous literature (Santos-Lozano et al., 2013, Sasaki et al., 2011).

Changes made: "To ensure that data reflected true physiological steady states, we adopted the protocol described in previous studies [8,19]. Thus, we calculated the mean activity counts from the four central minutes of the Vector Magnitude (VM) for GT3X+ and the counts (proportional integral mode - PIM) for ActTrust to estimate activity levels. METs (Metabolic Equivalents) were individually calculated (VO^2) similarly." (lines 119-124).

Minor Comment 4.7: Software version clarity

Reviewer: *Specify software and packages more clearly with version numbers.*

Response: We have included the package versions packages.

Changes Made: Revised to: "Analyses were conducted using R v. 4.4.1 (R Foundation for Statistical Computing, Vienna, Austria) [24] and auxiliary packages tidyverse v. 2.0.0 [25], caret v. 6.0.94 [26], MuMIn v. 1.48.4 [27], MetBrewer v. 0.2.0 [28], cowplot v. 1.1.3 [29], pROC v. 1.18.5 [30], broom v. 1.0.6 [31], lmtest v. 0.9.40 [32], car v. 3.1.2 [33], effectSize v. 1.0.2 [34], and msm v. 1.8.2 [35]. " (lines 140-142).

Minor Comment 4.8: ANOVA reporting

Reviewer: *ANOVA results mentioned but not fully reported. Consider including effect sizes.*

Changes Made: We have included the effect sizes: "We performed a two-way ANOVA to understand the outputs of each device (GT3X+ and ActTrust) according to the placement and condition (treadmill velocity). Movement counts from the GT3X+ devices were mostly distinct ($F(19, 1087) = 1347$, $p < 0.001$, 157 $\eta^2 = 0.96$, 95% CI [0.96, 1.00]) with

significantly lower activity magnitudes than those from the ActTrust devices at 3, 5, 7 and 9 km · h−1 ($p < 0.05$).” (lines 160-164).

Also note that the model was specified as an interaction-only term (Conditions:act), as the primary interest was in the combined effect of condition and device on movement counts; accordingly, we reported a single η^2 for this term.

Minor Comment 4.9: Figure referencing order

Reviewer: *Ensure all figures and tables are referenced in numerical order.*

Changes Made: We have verified and corrected the reference order.

Minor Comment 4.10: Data availability verification

Reviewer: *Ensure GitHub repository is well-documented and accessible.*

Response: We have enhanced the repository documentation.

Changes Made: We updated GitHub repository and included:

1. Comprehensive README.md with study overview, data structure explanation, and analysis instructions
2. Commented R scripts for all analyses
3. Data dictionary explaining all variables
4. Requirements.txt listing all software dependencies

We believe these comprehensive revisions have substantially strengthened the manuscript and fully addressed all reviewer concerns. We are grateful for the reviewers' expertise and hope the revised manuscript is now suitable for publication in PLOS ONE.

Sincerely,

Elias dos Santos Batista and colleagues
