# Supplementary material for: From movement to METs: A validation of ActTrust® for energy expenditure estimation and physical activity classification in young adults
Source: PLoS One. 2026 May 6;21(5):e0348631. doi: 10.1371/journal.pone.0348631 (PMC13148694; doi:10.1371/journal.pone.0348631)
Supplement: Attachment — Submitted filename: Responses to reviewers.pdf [file pone.0348631.s008.pdf]

Response to Reviewer Comments

Dear Editor,

We thank you and the reviewers for the thorough evaluation of our manuscript "From Movement to METs: A Validation of ActTrust® for Energy Expenditure Estimation and Physical Activity Classification in Young Adults".

We thank Reviewer #3 for their continued engagement with our manuscript and for confirming that the statistical analysis, data availability, and presentation now meet the standards required for publication in PLOS ONE.

In response to the reviewer's remaining comment regarding the distinction of biological sex in Figure 1, we have updated the figure accordingly. Point shape now encodes biological sex throughout both panels of Figure 1, with squares representing male participants and circles representing female participants, as suggested. This addition is reflected in the updated figure legend, which now reads: "Point shape denotes biological sex (square: male, n = 34; circle: female, n = 22)." We hope this provides the visual clarity the reviewer had in mind and offers the insight into the clustering observed at 7 $km \cdot h{-}1$that was noted in the initial review.

We believe this revision fully addresses the comment.

We thank the reviewer once more for their constructive and thorough engagement throughout the review process.

Sincerely,

Elias dos Santos Batista and colleagues
